# Mitochondrial imaging in live or fixed tissues using a luminescent iridium complex

**DOI:** 10.1038/s41598-018-24672-w

**Published:** 2018-05-29

**Authors:** Alexandra Sorvina, Christie A. Bader, Jack R. T. Darby, Mitchell C. Lock, Jia Yin Soo, Ian R. D. Johnson, Chiara Caporale, Nicolas H. Voelcker, Stefano Stagni, Massimiliano Massi, Janna L. Morrison, Sally E. Plush, Douglas A. Brooks

**Affiliations:** 1 Mechanisms in Cell Biology and Disease Research Group, School of Pharmacy and Medical Sciences, Sansom Institute for Health Research, University of South Australia, Adelaide, South Australia 5001 Australia; 2 Early Origins of Adult Health Research Group, School of Pharmacy and Medical Sciences, Sansom Institute for Health Research, University of South Australia, Adelaide, South Australia 5001 Australia; 3 Department of Chemistry and Curtin Institute for Functional Molecules and Interfaces, Curtin University, Bentley, Western Australia 6102 Australia; 4 Future Industries Institute, University of South Australia, Adelaide, South Australia 5095 Australia; 5 Drug Delivery, Disposition and Dynamics, Monash Institute of Pharmaceutical Sciences, Monash University, Parkville, Victoria, 3052 Australia; 6 Department of Industrial Chemistry “Toso Montanari”, University of Bologna, Bologna, I-40136 Italy

## Abstract

Mitochondrial morphology is important for the function of this critical organelle and, accordingly, altered mitochondrial structure is exhibited in many pathologies. Imaging of mitochondria can therefore provide important information about disease presence and progression. However, mitochondrial imaging is currently limited by the availability of agents that have the capacity to image mitochondrial morphology in both live and fixed samples. This can be particularly problematic in clinical studies or large, multi-centre cohort studies, where tissue archiving by fixation is often more practical. We previously reported the synthesis of an iridium coordination complex [Ir(**ppy**)_2_(**MeTzPyPhCN**)]^+^; where **ppy** is a cyclometalated 2-phenylpyridine and **TzPyPhCN** is the 5-(5-(4-cyanophen-1-yl)pyrid-2-yl)tetrazolate ligand; and showed that this complex (herein referred to as IraZolve-Mito) has a high specificity for mitochondria in live cells. Here we demonstrate that IraZolve-Mito can also effectively stain mitochondria in both live and fixed tissue samples. The staining protocol proposed is versatile, providing a universal procedure for cell biologists and pathologists to visualise mitochondria.

## Introduction

The normal function of muscle tissue is particularly reliant on mitochondria to fulfil high energy demand, to regulate calcium^1^ and to control ROS production^2^. Mitochondrial morphology is directly linked to many important cell and tissue functions, and consequently significant organelle remodelling is observed in response to changes in energy demand and cellular environment^3,4^. Changes in mitochondrial morphology are also observed in a range of human pathologies, including cardiovascular diseases and neuromuscular disorders^4–6^. Understanding the role of mitochondria in disease pathogenesis has been greatly advanced by the visualisation of these organelles, using a variety of microscopy techniques to image affected tissues^5,6^. Mitochondrial imaging by fluorescence microscopy is often utilised in medical research, but the currently available mitochondrial stains have mainly been limited to uses in live samples. This can be problematic for pathology testing, in clinical studies or in large cohort studies, where tissue samples cannot be immediately processed for assessment, and tissue preservation by fixation is highly preferable before imaging.

Mitochondrial imaging is primarily performed using fluorescence imaging by immunochemistry and small fluorescent molecules^7^. The majority of commercially available mitochondrial dyes are organic fluorophores that accumulate in the mitochondrial matrix due to the organelles transmembrane potential. These dyes are therefore only suited for use on live samples, for example, JC-1 and the MitoTrackers CMTMRos and CMXRos^8,9^. Commercial dyes that stain independently of mitochondrial polarisation tend to have an affinity for other mitochondrial-specific constituents (e.g. Mito-ID^®^ Red, which specifically binds to cardiolipin in the inner mitochondrial membrane), but their cellular uptake is still often limited to live samples^7^. To date, the visualisation of mitochondria in fixed samples has relied on immunochemistry. While antibody detection is sensitive, it is time consuming and requires multiple processing steps that may introduce significant artefacts. Moreover, issues with antibody availability can limit their use in a range of model species. There is, therefore, a need for small molecule imaging tools that can quickly and effectively image mitochondria in both live and fixed tissue samples.

Our laboratory has recently described the synthesis and characterisation^10^ of an iridium tetrazolato coordination complex [Ir(**ppy**)_2_(Me**TzPyPhCN**)]^+^, where **ppy** is a cyclometalated 2-phenylpyridine and **TzPyPhCN** is the 5-(5-(4-cyanophen-1-yl)pyrid-2-yl)tetrazolate ligand (Supplementary Fig. 1). This iridium complex is commercially available as IraZolve-Mito and exhibits a high specificity for mitochondria in live H9c2 rat cardiomyoblasts^10^. The use of iridium complexes, and other transition metal complexes as imaging agents has gained increasing attention due to their superior photostability, large Stoke shifts and long excited states, when compared to organic fluorophores^11^. Several iridium complexes demonstrate localisation to mitochondria^12–15^, but to the best of our knowledge, their utility for mitochondrial detection in fixed tissue samples has not yet been explored. Recognising this need for new mitochondrial stains that are compatible with fixed tissue samples, we have provided validation of a protocol for the visualisation of mitochondria using IraZolve-Mito in both live and fixed tissues by confocal microscopy.

## Results

**Figure 1:**
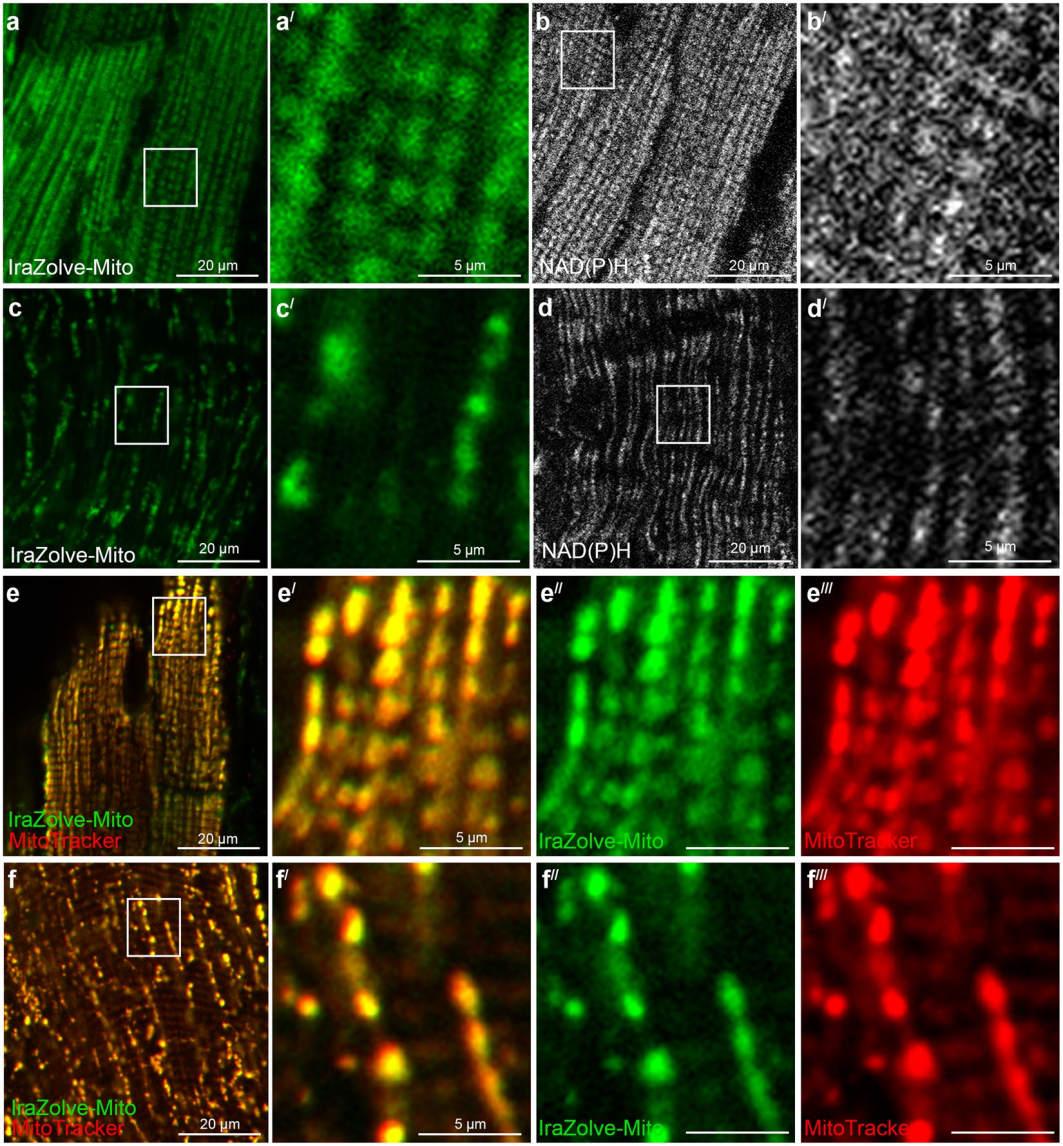
IraZolve-Mito detects mitochondria in live cardiac and skeletal muscle samples. Representative confocal micrographs showing mitochondria detected with IraZolve-Mito in cardiac (**a**; enlarged in **a**^**/**^) and skeletal (**c**; enlarged in **c**^**/**^) muscle samples. Endogenous NAD(P)H detected by two-photon microscopy in cardiac (**b**; enlarged in **b**^**/**^) and skeletal (**d**; enlarged in **d**^**/**^) muscle samples (excited at 740 nm and collected at 474–504 nm). Micrographs showing co-staining of mitochondria with IraZolve-Mito (green in **e–e**^**//**^ and **f**–**f**^**//**^) and MitoTracker Red CMXRos (red in **e**,**e**^**/**^,**e**^**///**^ and **f**,**f**^**/**^,**f**^**///**^) in cardiac (**e**; enlarged in **e**^**/**^–**e**^**///**^) and skeletal (**f**; enlarged in **f**^**/**^–**f**^**///**^) muscle samples. Scale bars: 20 µm (**a**–**f**) and 5 µm (**a**^**/**^–**d**^**/**^, **e**^**/**^–**e**^**///**^ and **f**^**/**^–**f**^**///**^).

The localisation of IraZolve-Mito was assessed in live, fresh frozen and fixed muscle tissues. Muscle tissues were selected due to their high abundance of mitochondria and clinical significance for altered mitochondrial morphology^4–6^. Tissue samples were collected from the left ventricle of the heart (cardiac muscle) and quadriceps (skeletal muscle) of adult sheep. For live imaging, tissue samples were cut into ~5 mm cubes and incubated for 30 minutes at room temperature with 20 µM of IraZolve-Mito in 0.2% (v/v) DMSO/PBS. After this incubation, the tissues were washed for 5 minutes before mounting and imaging on a Nikon A1^+^ confocal microscope. As previously reported, when excited at 403 nm, IraZolve-Mito exhibited a broad emission band with an emission maximum at ~608 nm^10^. Images were therefore acquired in the emission range 505–625 nm. In live cardiac and skeletal muscle, IraZolve-Mito stained cylindrical-shaped organelles (Fig. 1a,a^/^,c,c^/^), which resembled mitochondria. These organelles were observed in close proximity to sarcomeres (Fig. 1a,a^/^) and arranged in a regular network structure throughout the muscle fibres (Fig. 1a,c). This distinctive distribution was consistent with previous observations of mitochondria in skeletal muscle^16,17^.

The distribution of NAD(P)H in live tissue was detected using two-photon microscopy for comparison with IraZolve-Mito staining in live tissue. NAD(P)H is known to be associated with mitochondria^18^ and produces a strong endogenous fluorescence at 489 nm (474–504 nm emission interval), when exposured to two-photon illumination at 740 nm^19^. The distribution of NAD(P)H (Fig. 1b,b^/^,d,d^/^) was comparable to the staining pattern of IraZolve-Mito (Fig. 1a,a^/^,c,c^/^). To further validate the localisation of IraZolve-Mito to mitochondria, live samples were co-stained with MitoTracker. There was a significant co-location of these dyes in both cardiac muscle (Pearson’s correlation 0.84 ± 0.01, n = 6; Fig. 1e–e^///^) and skeletal muscle (Pearson’s correlation 0.86 ± 0.03, n = 7; Fig. 1f–f^///^), confirming the localisation of IraZolve-Mito to mitochondria, as previously observed in live cells^10^.

**Figure 2:**
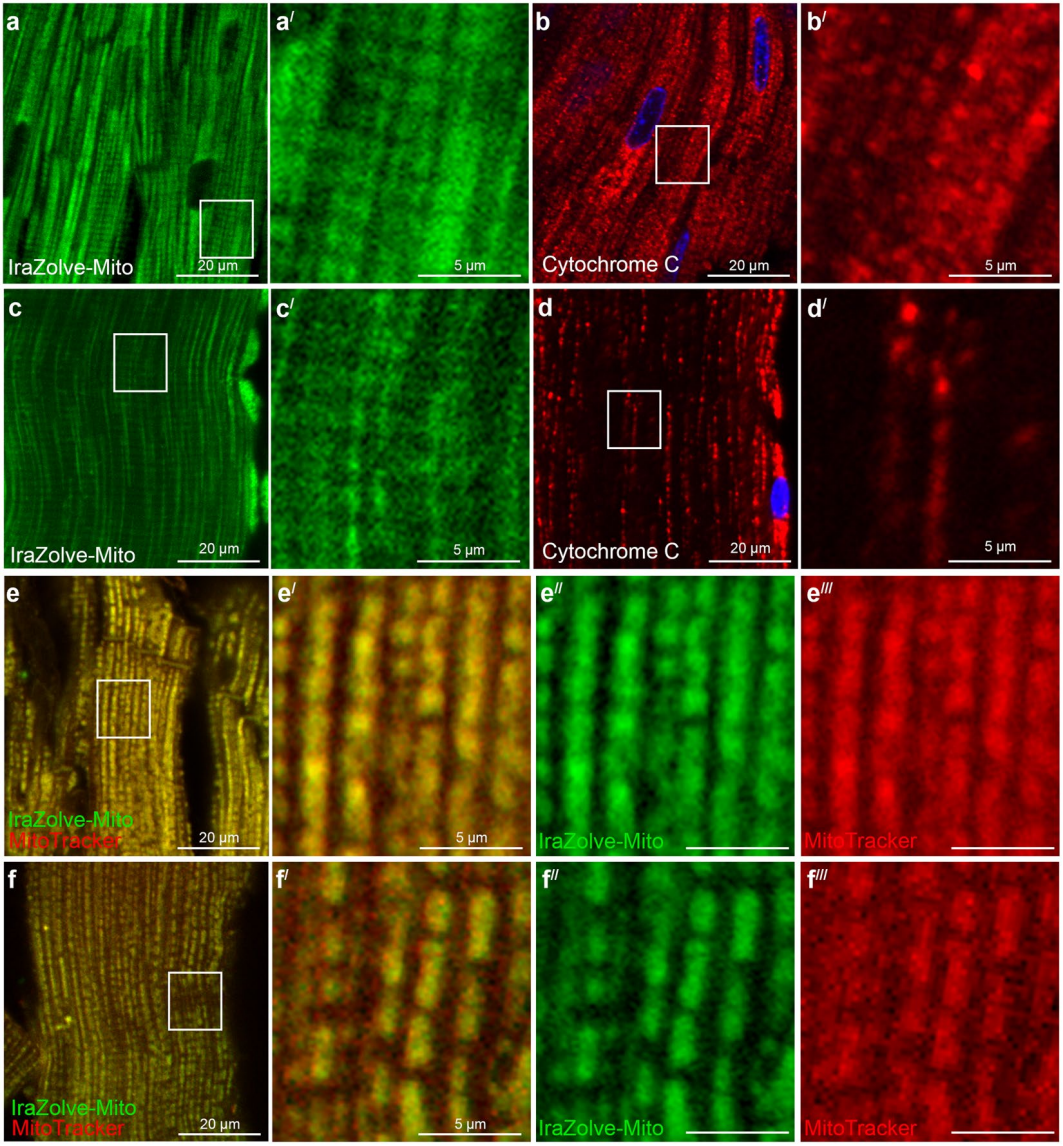
Staining pattern of IraZolve-Mito is maintained in fixed cardiac and skeletal muscle samples. Representative confocal micrographs showing subcellular distribution of IraZolve-Mito in 4% PFA fixed cardiac (**a**; enlarged in **a**^**/**^) and skeletal (**c**; enlarged in **c**^**/**^) muscle samples. Confocal micrographs showing localisation of Cytochrome C detected with anti-Cytochrome C antibody in cardiac (**b** enlarged in **b**^**/**^) and skeletal (**d**; enlarged in **d**^**/**^) muscle samples. Micrographs showing co-staining of mitochondria with IraZolve-Mito (green in **e–e**^**//**^ and **f–f**^**//**^) and MitoTracker Red CMXRos (red in **e**,**e**^**/**^,**e**^**///**^ and **f**,**f**^**/**^,**f**^**///**^) in cardiac (**e**; enlarged in **e**^**/**^–**e**^**///**^) and skeletal (**f**; enlarged in **f**^**/**^–**f**^**///**^) muscle samples. Scale bars: 20 µm (**a**–**f**) and 5 µm (**a**^**/**^–**d**^**/**^, **e**^**/**^–**e**^**///**^ and **f**^**/**^–**f**^**///**^).

We next assessed IraZolve-Mito staining using fixed cardiac and skeletal muscle tissues. These tissues were fixed in 4% paraformaldehyde (PFA) for ~20 hours and after a 30 minute wash step, were stored in PBS at 4 °C. For these fixed samples, the IraZolve-Mito staining and visualisation was performed using the same protocol that had been optimised for live tissues. The IraZolve-Mito staining pattern in fixed tissue (Fig. 2a,a^/^,c,c^/^**)** mirrored that observed for live muscle samples (Fig. 1a,a^/^,c,c^/^). Further the staining pattern was consistent with antibody probing for cytochrome C protein, which localises to the inner membrane of mitochondria (Fig. 2b,b^/^,d,d^/^). For confirmation live tissue samples were stained with MitoTracker and, after fixation in 4% PFA, counter stained with IraZolve-Mito. For this procedure, the tissue fixation was performed for 1 hour to prevent the loss of MitoTracker, but this was sufficient for the fixation of these small samples (≤5 mm thickness). The co-location of IraZolve-Mito and MitoTracker in fixed cardiac muscle (Pearson’s correlation 0.89 ± 0.01, n = 4; Fig. 2e–e^///^) and skeletal muscle (Pearson’s correlation 0.89 ± 0.02, n = 3; Fig. 2f–f^///^) demonstrated the capacity of IraZolve-Mito to image mitochondria in PFA fixed tissue.

To investigate the scope of the applications for IraZolve-Mito, co-localisation was also attempted by combining IraZolve-Mito staining with anti-Cytochrome C antibody staining, but unfortunately the specificity of IraZolve-Mito was lost in this process (Supplementary Fig. 2). Although some specific staining could be maintained when protocols were adjusted to include short permeabilisation, these protocols yielded less than desirable staining for IraZolve-Mito, and we concluded that IraZolve-Mito was less than ideal when used in combination with immunofluorescence. Alternative tissue preparations were also assessed for compatibility with IraZolve-Mito including formalin-fixed paraffin-embedded samples and flash frozen samples. The staining pattern of IraZolve-Mito was not maintained in formalin-fixed paraffin-embedded skeletal muscle tissues both with and without antigen retrieval protocols (Supplementary Fig. 3). However, tissue samples that were flash frozen did show staining consistent with mitochondrial using the presented protocol (Supplementary Fig. 4). This demonstrates that IraZolve-Mito provides a tool for imaging mitochondria in samples prepared for storage by PFA fixation or flash freezing, without the need to stain while the tissue is alive.

**Figure 3:**
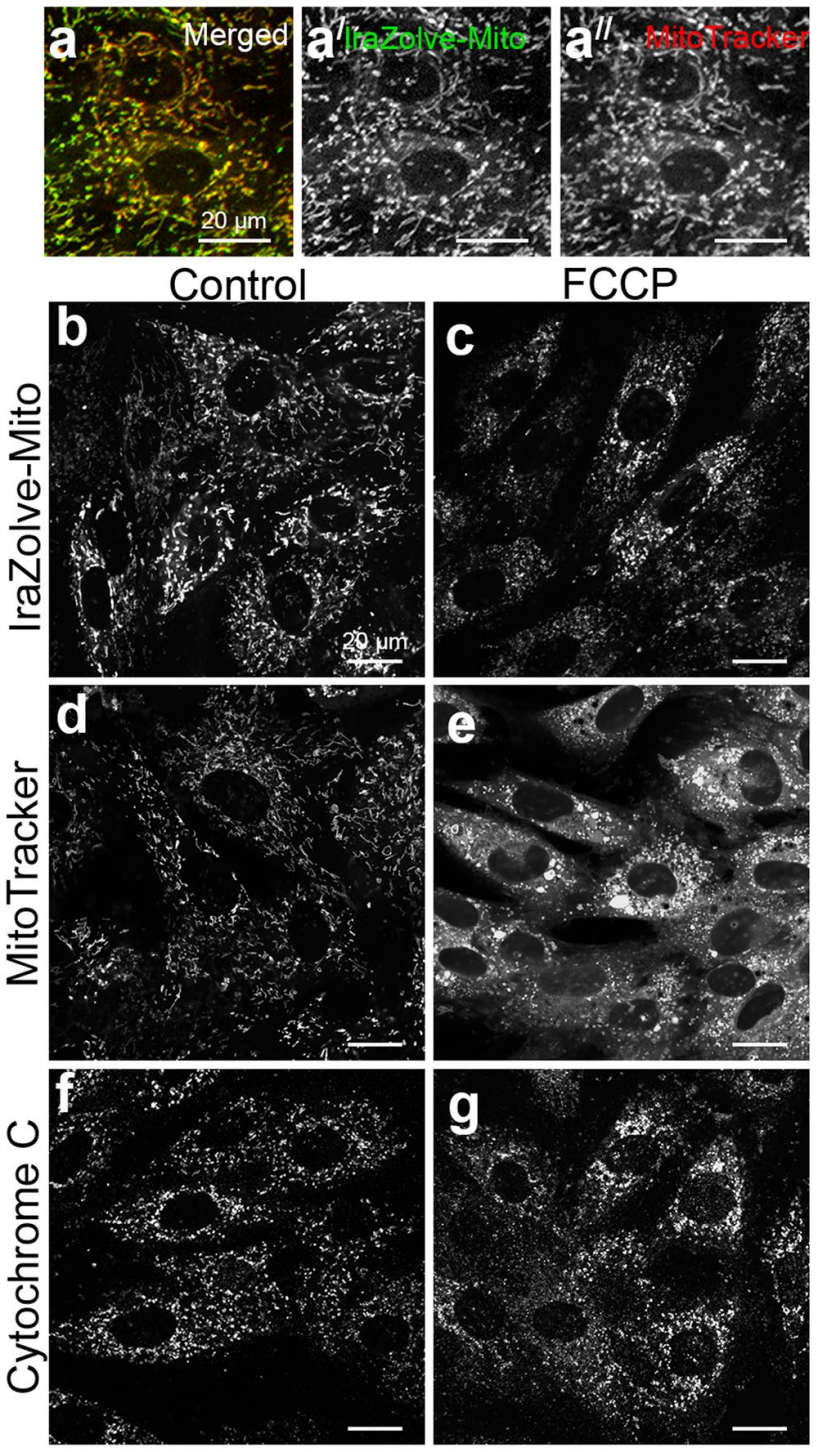
Inhibition of mitochondrial membrane potential in H9c2 rat cardiomyoblasts does not disrupt IraZolve-Mito staining. (**a**–**a**^**//**^) Micrographs showing co-staining of IraZolve-Mito (green in **a**; greyscale in **a**^**/**^) with MitoTracker Red CMXRos (red in **a**; greyscale in **a**^**//**^) in cardiomyoblasts. Scale bars: 20 µm. Cardiomyoblasts were stained with IraZolve-Mito (**b**,**c**), MitoTracker Red CMXRos (**d**,**e**) or by anti-Cytochrome C antibody (**f**,**g**) either under normal conditions (control; **b**,**d**,**f**) or following treatment with FCCP (**c**,**e**,**g**). Scale bars: 20 µm.

The compatibility of IraZolve-Mito for mitochondrial staining of fixed tissue suggests that the mechanism controlling localisation is not solely based on its cationic nature. To confirm that the localisation of IraZolve-Mito with mitochondria was not dependent on membrane polarisation, we deregulated the mitochondrial membrane potential using carbonyl cyanide-*p*-trifluoromethoxyphenylhydrazone (FCCP)^20^. In H9c2 rat cardiomyoblasts FCCP treatment did not perturb IraZolve-Mito mitochondrial staining. By comparison MitoTracker, which is dependent on mitochondrial membrane potential^8,9^, exhibited dispersed cytoplasmic staining in FCCP treated cells (Fig. 3). Cytochrome C immunofluorescence confirmed that there were morphological changes to mitochondria following FCCP treatment which match IraZolve-Mito staining patterns (Fig. 3c,g). This indicated that IraZolve-Mito staining was not reliant on membrane polarisation for mitochondrial accumulation.

## Discussion

IraZolve-Mito provides an alternative tool for the visualisation of mitochondria in both live and fixed tissue samples, which is quick and easy to use. The labelling of mitochondria by IraZolve-Mito was compared to other available imaging technologies including live cell staining by MitoTracker, endogenous fluorescence from NAD(P)H and antibody staining by anti-Cytochrome C. The ability of IraZolve-Mito to stain tissue that has been fixed by PFA or flash frozen allows for a simple approach to imaging mitochondria in samples that are suitable for long term storage. This provides a tool to retrospectively interrogate mitochondrial localisation and morphology in tissues collected, for example, in clinical or preclinical trails, without the need for pre-emptive staining during tissue collection or for time consuming immunochemistry protocols after tissue collection. The staining of mitochondria in fixed samples has not previously been possible without the use of immunochemistry. Immunochemistry protocols have some significant limitations: including the size of the antibody molecule that can impair their ability to penetrate the cell and tissue sample; the specificity/reproducibility of the primary and secondary antibody detection reagents; availability of species specific primary antibodies and the lengthy, multistep processes that can introduce significant artefacts. These immunochemistry protocols can also be costly and time consuming, when compared to small molecule imaging technologies like IraZolve-Mito. IraZolve-Mito offers an easy to use mitochondrial marker that is not dependent on protein localisation, which may be altered in certain physiological conditions.

Unlike the majority of mitochondrial dyes IraZolve-Mito was not reliant on mitochondrial membrane potential for localisation, which likely accounts for its compatibility with fixed tissue staining. This gives a distinct advantage for the use of IraZolve-Mito by allowing the visualisation of mitochondria during conditions of stress for live cells and tissues which may be induced by drug treatments or pathological processes. A number of similar cationic iridium complexes have shown membrane potential independent localisation to the mitochondria^12–15,21,22^. This would indicate that this family of molecules has an alternative targeting mechanism, possibly via protein or lipid association. Investigation of one cationic iridium complex, Ir-ITC, demonstrated that this complex had an association with mitochondrial proteins including transmembrane protein, VDAC1 (voltage-dependent anion channel 1)^23^. In this study staining was carried out in live cells, and the resulting association of Ir-ITC may be a consequence of the favourable conditions within the mitochondria, which accelerates the rate of bond formation between the reactive isothiocyanate unit of the Ir-ITC and amines, as opposed to the targeting ability of the complex^23^. The observed loss of specificity of staining by IraZolve-Mito in samples that have undergone dewaxing (paraffin-embed samples) or permeabilisation, which typically remove lipids^24,25^, suggested a mechanism that may involve lipid association. Moreover, IraZolve-Mito and other mitochondria localising iridium complexes are commonly lipophilic^12–15,21,22^. Though the molecular target for IraZolve-Mito has yet to be identified, increased access to technologies such as super-resolution microscopy may provide critical insight into submitochondrial localisation and aid in the identification of the target.

To the best of our knowledge, this capacity of IraZolve-Mito to image mitochondria in both live and fixed tissues makes this compound unique and the first of its kind for a small molecule that has the potential to easily penetrate and image mitochondria in fixed tissues. As the localisation is not dependent on membrane potential or protein localisation, IraZolve-Mito is broadly applicable to tissue samples, even under conditions of mitochondrial stress that may cause membrane depolarisation or protein relocation. The ability to stain fixed tissue also provide researchers with an easy to use alternative to antibody staining and which does not require the foresight of staining live tissues during collection. The identification of small luminescent molecules, such as IraZolve-Mito, that is suitable for imaging mitochondria in fixed tissue samples and that is ideal for live cell and tissue imaging, provides researchers with greater flexibility in experimental design and sample preparation and reduces the need for multiple reagents.

## Conclusions

The iridium coordination complex IraZolve-Mito is an effective imaging agent for mitochondria, and is suitable for use on live, frozen and PFA fixed tissue, as well as for live cell cultures. Therefore, IraZolve-Mito provides much greater flexibility for mitochondrial imaging, with potential utility in large cohort studies or within clinical settings where the use of live tissue samples is logistically challenging.

## Methods

### Chemical synthesis and photophysical properties of IraZolve-Mito

Experimental details and characterisation for IraZolve-Mito (previously reported as [Ir(ppy)2(MeTzPyPhCN)]+) can be found in Caporale *et al*.^10^.

### Animal procedures and muscle samples

The experimental procedures were approved by the South Australian Health and Medical Research Institute Animal Ethics Committee and followed the guidelines of the Australian Code of Practice for the Care and Use of Animals for Scientific Purposes developed by the National Health and Medical Research Council. The investigators understood the ethical principles outlined in Grundy *et al*.^26^. Adult pregnant ewes (4 yrs old; n = 4) were housed in an individual pen in view of other sheep in an indoor housing facility that was maintained at a constant ambient temperature of between 20–22 °C and a 12 hour light/dark cycle. Sheep were humanely killed via overdose of sodium pentobarbitone (8 g; Vibrac Australia, Peakhurst, Australia). Samples of cardiac muscle tissue were taken from the left ventricle and skeletal muscle was taken from the quadriceps. Collected tissue samples were placed into sterile phosphate-buffered saline solution (PBS; Sigma-Aldrich, St. Louis, USA) on ice, protected from light, transported to the imaging facility within 90 minutes and imaged within 7 hours. All experiments were performed on samples from a minimum of 4 animals on 3 separate days.

### Preparation of live tissue sections

Live tissue samples were cut using a sharp scalpel to allow clean cutting and prevent damage associated with tearing of tissue. Sections were no more than 5 mm in thickness. The sectioning was performed in sterile PBS at room temperature (21 ± 2 °C).

### Preparation of paraformaldehyde fixed tissue sections

After dissection, cardiac (~1 cm^3^) and skeletal muscle (~1 cm^3^) tissues were washed in PBS, and then submerged in 4% paraformaldehyde (PFA; Sigma-Aldrich, St. Louis, USA) for 20 hours at 4 °C. PFA was removed and the fixed tissues were washed in PBS for 30 minutes at room temperature. Samples were then stored in PBS at 4 °C. Fixed tissue sections of ~2 mm thickness were cut by using a sharp scalpel in sterile PBS. Prior to staining, tissue sections were kept in PBS for 2 hours at room temperature.

### Preparation of frozen tissue sections

Samples of skeletal muscle were snap frozen in liquid nitrogen and stored at −80 °C. Sections were cut on a Leica CM1950 clinical cryostat (Leica Biosystems, Australia) at 5 µm-thick and collected onto SuperFrost PLUS charged glass slides (Menzel-Gläser, Germany). Tissue sections were heat-fixed to slides at 60 °C for at least 60 minutes and slides were stored at −20 °C until required. Cryosections were thawed at room temperature for 30 minutes, before being placed in PBS for 5 minutes for rehydration.

### Tissue staining

A staining solution of IraZolve-Mito (ReZolve Scientific, Adelaide, Australia) was prepared from a 10 mM stock (prepared in DMSO; Sigma-Aldrich, St. Louis, USA), which was diluted in sterile PBS to a final concentration of 20 µM (0.2% DMSO). Fresh and PFA fixed tissues were fully submerged in 1 mL of the staining solution in 5 mL tubes and incubated at room temperature with gentle agitation provided by a rocker for 30 minutes. The staining solution was aspirated and tissues were then washed for 5 minutes in PBS. For mitochondrial staining by MitoTracker Red CMXRos (diluted at 1:1000 in PBS; Life Technologies Australia Pty Ltd., Mulgrave, Australia) tissues were fully immersed in 1 mL of staining solution and incubated on ice for 15 minutes with general agitation^27^. Tissues were then washed in PBS for 5 minutes. For anti-Cytochrome C antibody (Sapphire Bioscience, Redfern, Australia) probing, PFA fixed tissues were permeabilised with 0.1% Saponin (Sigma-Aldrich, St. Louis, USA) in PBS for 2 hours at room temperature. To block non-specific binding of the antibodies, tissues were submerged in 5% bovine serum albumin (BSA; Sigma-Aldrich, St. Louis, USA) containing 0.05% Saponin for 2 hours at room temperature. Tissues were then incubated with anti-Cytochrome C antibody (1 µg/mL; prepared in 5% BSA containing 0.05% Saponin) overnight with gentle agitation provided by a rocker platform incubated at 4 °C. After a washing step, secondary anti-IgG antibody conjugated with Cy5 labels (Jackson ImmunoResearch Laboratories, West Grove, USA) were then added and tissues were incubated for an hour at room temperature before a final wash step was performed. These washing steps were performed for 2 hours at room temperature. The Hoechst 33258 DNA stain (1:1000 in PBS; Life Technologies Australia Pty Ltd., Mulgrave, Australia) was performed for 1 minute, and followed by a 5 minute wash in PBS.

### Mounting tissues for imaging

For tissue mounting, µ-slide 8 well chambers (DKSH, Hallam, Australia) were used. Tissues were kept moist, but were not mounted in PBS as this caused tissues to float away from the imaging surface. Tissues were mounted with a longitudinal cross-section in contact with the imaging surface of the chamber, where possible. Good contact between the sample and the surface was achieved by gently pressing the tissue down into the chamber. Using samples that were roughly the same size as the chamber, also helped to hold the tissue in place. A dissection microscope was used to place and orientate the tissues.

### Imaging by confocal and two-photon microscopy

Once mounted, tissues were immediately imaged with no more than 30 minutes elapsing between mounting and image completion, as imaging beyond this time can lead to excessive drying of tissue samples. Stained tissues were imaged using a Nikon A1+(Nikon, Japan), fitted with a LU-N4/LU-N4S 4-laser unit (403, 488, 561 and 640 nm), the A1-DUG GaAsP Multi Detector Unit (2 GaAsP PMTs + 2 standard PMTs) and a 32 channel spectral detector (Nikon, Minato, Tokyo, Japan). Images for IraZolve-Mito were collected using a 403 nm laser set to 2 power setting and emission between 505 and 625 nm detected by the spectral detector, with gain set to 180 for cardiac tissue and 170 for skeletal muscle tissue. The pinhole radius was 42.1 µm. Unstained muscle samples were used as negative controls to confirm that the settings used for the visualisation of IraZolve-Mito did not detect endogenous fluorescence (Supplementary Fig. 5). For imaging of MitoTracker Red CMXRos, 561 nm excitation wavelength (0.3 power setting) was used and emission was collected at 595 nm by a GaAsP PMT detector (gain of PMT HV 30). For co-staining experiments the settings above were used in sequence to collect IraZolve-Mito and MitoTracker Red CMXRos respectively, minimising any overlap in spectral profiles. For co-staining experiments the settings above were used in sequence to collect IraZolve-Mito and MitoTracker Red CMXRos respectively, minimising any overlap in spectral profiles. Note that prior to co-staining experiments, the possible spectral crosstalk between IraZolve-Mito and MitoTracker Red CMXRos was evaluated in tissue samples by assessing the spectra for each dye excited when excited with 403 nm and 561 nm lasers. Anti-Cytochrome C antibody staining was imaged using a 640 nm laser (7 power setting) and emission wavelength 700 nm by a standard PMT (gain of PMT HV 125). All images were captured using a 40x/WI λS DIC N2 water emersion lens.

For the detection of NAD(P)H in fresh tissues, a Zeiss LSM710 NLO confocal microscope equipped with a two-photon Mai-Tai, tunable Ti:Sapphire femtosecond pulse laser (Spectra-Physics, USA) was utilised. Images were collected using two-photon excitation at 740 nm and a 474–504 nm emission interval^19^. The laser power was 11% andthe pinhole set to 600 µm. The pixel dwell time was set to 1.58 µs, and each image was averaged eight times to increase the signal-to-noise ratio. All images were acquired using a LD C-Apochromat 40x/NA 1.1 Water Corr UV-VIS-IR M27 objective (Carl Zeiss, Jena, Germany). The temperature in the imaging facility was set to a constant 19 °C.

### Image analysis

All images were assembeled using Adobe Photoshop CS6 (ver. 13.0 × 64; Microsoft, USA). For analysis of co-localisation by Pearson Correlations, NIS-elements analysis software (Nikon, Japan), was utilised.

### Inhibition of membrane potential

To investigate if cellular localisation of IraZolve-Mito was driven by mitochondrial membrane potential, H9c2 rat cardiomyoblast cells were treated with carbonyl cyanide-p-trifluoromethoxyphenylhydrazone (FCCP; Sigma-Aldrich, St. Louis, USA)^20^. The H9c2 cells (1 × 10^5^ cells/mL) were plated in ibidi µ-slide 8 wells and cultured overnight at 37 °C and 5% CO_2_ in 250 µL of DMEM medium (Sigma-Aldrich, St. Louis, USA) supplemented with 10% fetal bovine serum (FBS; *In Vitro* Technologies, USA) and 2 mM L-glutamine (Sigma-Aldrich, St. Louis, USA). The cells were treated with FCCP, prepared in a complete DMEM media at 100 μM concentration, for 30 minutes at 37 °C and 5% CO_2_. Media was then aspirated and the H9c2 cells were either fixed in 4% PFA for 30 minutes to be used for antibody staining or stained with either [Ir(ppy)2(MeTzPyPhCN)]+ (20 µM) or MitoTracker (1:1000) for 30 minutes in the presence of FCCP. After staining, the cells were washed and then 300 µL of cell culture media was added for imaging on Nikon A1+ microscope.

## Electronic supplementary material

Supplementary Figures

## References

[1] Rizzuto R, De Stefani D, Raffaello A, Mammucari C (2012). Mitochondria as sensors and regulators of calcium signalling. Nat Rev Mol Cell Biol.

[2] Starkov AA (2008). The role of mitochondria in reactive oxygen species metabolism and signaling. Ann N Y Acad Sci.

[3] Liesa M, Shirihai OS (2013). Mitochondrial dynamics in the regulation of nutrient utilization and energy expenditure. Cell Metab.

[4] Dorn GW (2015). Mitochondrial dynamism and heart disease: changing shape and shaping change. EMBO Mol Med.

[5] Miller N, Shi H, Zelikovich AS, Ma YC (2016). Motor neuron mitochondrial dysfunction in spinal muscular atrophy. Hum Mol Genet.

[6] Lopez-Crisosto, C. *et al*. Sarcoplasmic reticulum-mitochondria communication in cardiovascular pathophysiology. *Nat*. *Rev*. *Cardiol* (2017). 10.1038/nrcardio.2017.23 28275246

[7] Cottet-Rousselle C, Ronot X, Leverve X, Mayol JF (2011). Cytometric assessment of mitochondria using fluorescent probes. Cytometry. Part A.

[8] Chen CS, Gee KR (2000). Redox-dependent trafficking of 2,3,4,5, 6-pentafluorodihydrotetramethylrosamine, a novel fluorogenic indicator of cellular oxidative activity. Free Radic Biol Med.

[9] Cossarizza A, Baccarani-Contri M, Kalashnikova G, Franceschi C (1993). A new method for the cytofluorimetric analysis of mitochondrial membrane potential using the J-aggregate forming lipophilic cation 5,5′,6,6′-tetrachloro-1,1′,3,3′-tetraethylbenzimidazolcarbocyanine iodide (JC-1). Biochem. Biophys. Res. Commun..

[10] Caporale, C. *et al*. Investigating intracellular localisation and cytotoxicity trends for neutral and cationic iridium tetrazolato complexes in live cells. *Chem*. *Eur*. *J* (2017). 10.1002/chem.201701352 28782852

[11] Lo KK (2015). Luminescent rhenium(I) and iridium(III) polypyridine complexes as biological probes, imaging reagents, and photocytotoxic agents. Acc Chem Res.

[12] Huang H (2016). Real-time tracking mitochondrial dynamic remodeling with two-photon phosphorescent iridium (III) complexes. Biomaterials.

[13] Sun L (2016). Iridium(III) anthraquinone complexes as two-photon phosphorescence probes for mitochondria imaging and tracking under hypoxia. Chemistry.

[14] Chen Y, Qiao L, Ji L, Chao H (2014). Phosphorescent iridium(III) complexes as multicolor probes for specific mitochondrial imaging and tracking. Biomaterials.

[15] Chen Y (2013). Mitochondria-specific phosphorescent imaging and tracking in living cells with an AIPE-active iridium(III) complex. ChemComm (Cambridge, England).

[16] Glancy B (2015). Mitochondrial reticulum for cellular energy distribution in muscle. Nature.

[17] Vendelin M (2005). Mitochondrial regular arrangement in muscle cells: a “crystal-like” pattern. Am J Physiol-Cell Ph.

[18] Lewis CA (2014). Tracing compartmentalized NADPH metabolism in the cytosol and mitochondria of mammalian cells. Mol Cell.

[19] Sorvina, A. *et al*. Label-free imaging of healthy and infarcted fetal sheep hearts by two-photon microscopy. *J*. *Biophotonics* (2017). 10.1002/jbio.201600296 28464439

[20] Kalbacova M, Vrbacky M, Drahota Z, Melkova Z (2003). Comparison of the effect of mitochondrial inhibitors on mitochondrial membrane potential in two different cell lines using flow cytometry and spectrofluorometry. Cytometry A.

[21] Chen Y, Xu WC, Zuo JR, Ji LN, Chao H (2015). Dinuclear iridium(III) complexes as phosphorescent trackers to monitor mitochondrial dynamics. J Mater Chem B.

[22] Qiu KQ (2015). Mitochondria-specific imaging and tracking in living cells with two-photon phosphorescent iridium(III) complexes. J Mater Chem B.

[23] Wang B (2012). A luminescent cyclometalated iridium(III) complex accumulates in mitochondria and induces mitochondrial shortening by conjugation to specific protein targets. Chembiochem.

[24] Hobro AJ, Smith NI (2017). An evaluation of fixation methods: Spatial and compositional cellular changes observed by Raman imaging. Vib Spectrosc.

[25] Oliver C, Jamur MC (2010). Immunocytochemical methods and protocols. Methods in molecular biology (Clifton, N.J.).

[26] Grundy D (2015). Principles and standards for reporting animal experiments in The Journal of Physiology and Experimental Physiology. J. Physiol..

[27] Johnson S, Rabinovitch P (2012). *Ex vivo* imaging of excised tissue using vital dyes and confocal microscopy. Current protocols in cytometry.

