# Supplementary material for: Mitochondrial imaging in live or fixed tissues using a luminescent iridium complex
Source: Sci Rep. 2018 May 29;8:8191. doi: 10.1038/s41598-018-24672-w (PMC5974328; doi:10.1038/s41598-018-24672-w)
Supplement: Supplementary file 1 — Supplementary Figures [file 41598_2018_24672_MOESM1_ESM.docx]

**Mitochondrial imaging in live or fixed tissues using a luminescent iridium complex**

**Alexandra Sorvina^1^, Christie A. Bader^1^, Jack R. T Darby^2^, Mitchell C. Lock^2^, Jia Yin Soo^2^, Ian R. D. Johnson^1^, Chiara Caporale^3^, Nicolas H. Voelcker^4,5^, Stefano Stagni^6^, Massimiliano Massi^3,1^, Janna L. Morrison^2^, Sally E. Plush^1,4^ and Douglas A. Brooks^1^**

^1^Mechanisms in Cell Biology and Disease Research Group, School of Pharmacy and Medical Sciences, Sansom Institute for Health Research, University of South Australia, Adelaide, South Australia 5001, Australia.

^2^Early Origins of Adult Health Research Group, School of Pharmacy and Medical Sciences, Sansom Institute for Health Research, University of South Australia, Adelaide, South Australia 5001, Australia.

^3^Department of Chemistry and Curtin Institute for Functional Molecules and Interfaces, Curtin University, Bentley, Western Australia 6102, Australia.

^4^Future Industries Institute, University of South Australia, Adelaide, South Australia, 5095, Australia.

^5^Drug Delivery, Disposition and Dynamics, Monash Institute of Pharmaceutical Sciences, Monash University, Parkville, Victoria 3052, Australia.

^6^Department of Industrial Chemistry “Toso Montanari”, University of Bologna, Bologna I-40136, Italy.

Correspondence should be addressed to S. E. P. ([Sally.Plush@unisa.edu.au](mailto:Sally.Plush@unisa.edu.au)) or D.A.B. ([Doug.Brooks@unisa.edu.au](mailto:Doug.Brooks@unisa.edu.au))

**Supplementary Information**


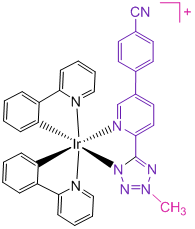


**Supplementary Figure 1|** Structure of IraZolve-Mito.


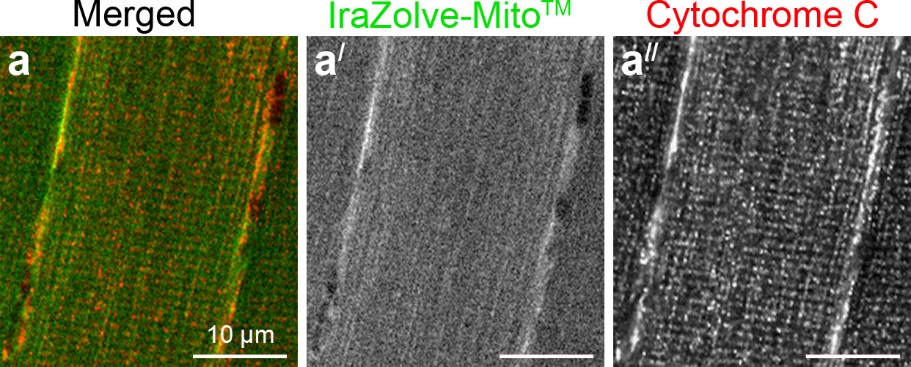


**Supplementary Figure 2|** Co-staining of IraZolve-Mito with antibody resulted in only faint staining with the former. Confocal micrographs showing subcellular localisation of IraZolve-Mito (green in **a**; greyscale in **a^/^**) in relation to Cytochrome C (red in **a**; greyscale in **a^//^**) in fixed skeletal muscle samples. Scale bars: 10 µm.

Staining protocol for paraffin embedded samples

Formalin-fixed paraffin-embedded skeletal muscle tissue sections (5 µm thick) were dewaxed in xylene, rehydrated in a graded series of ethanol concentrations and then separated in two groups, one of which underwent heat-induced antigen retrieval at pH 6. Sections were stained with IraZolve-Mito for 30 minutes at room temperature and then washed for 5 minutes in PBS. As tissues exhibited strong fluorescence, spectral unmixing procedure was applied for the detection of IraZolve-Mito. Images for IraZolve-Mito were obtained using a 403 nm laser and emission was collected at 608 nm (emission wavelength bandwidth was set to 89.8 nm). Although it was possible to detect IraZolve-Mito in formalin-fixed paraffin-embedded skeletal muscle samples, the staining pattern of this imaging reagent was not maintained.


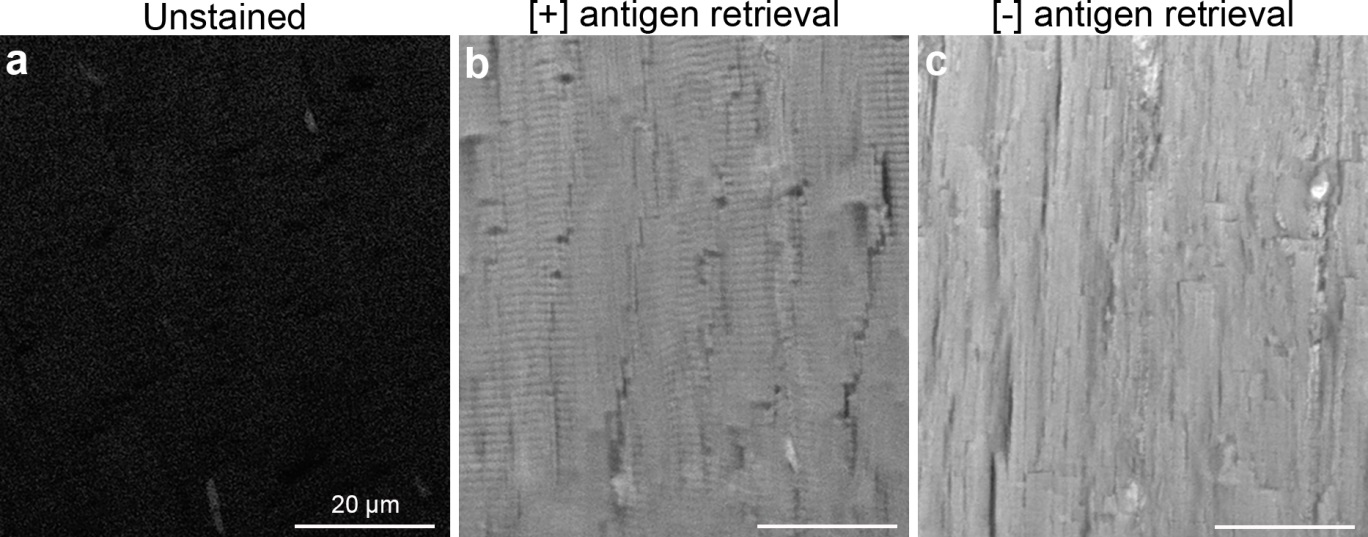


**Supplementary Figure 3|** Staining pattern of IraZolve-Mito is not maintained in formalin-fixed paraffin-embedded (FFPE) skeletal muscle samples. Representative confocal micrographs showing unstained (**a**) and stained with IraZolve-Mito (**b**, **c**) FFPE skeletal muscle samples. Scale bars: 20 µm.


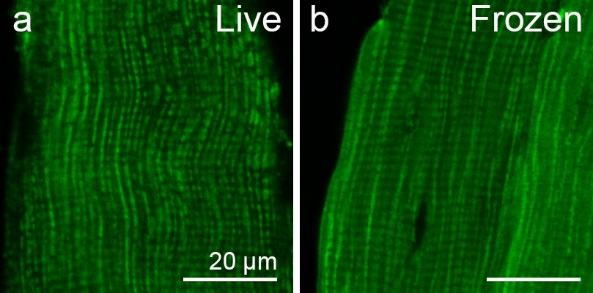


**Supplementary Figure 4|** Staining pattern of IraZolve-Mito is maintained in frozen skeletal muscle samples. Representative confocal micrographs showing subcellular distribution of IraZolve-Mito in live (**a**) and frozen (**b**) skeletal muscle samples. Scale bars: 20 µm.


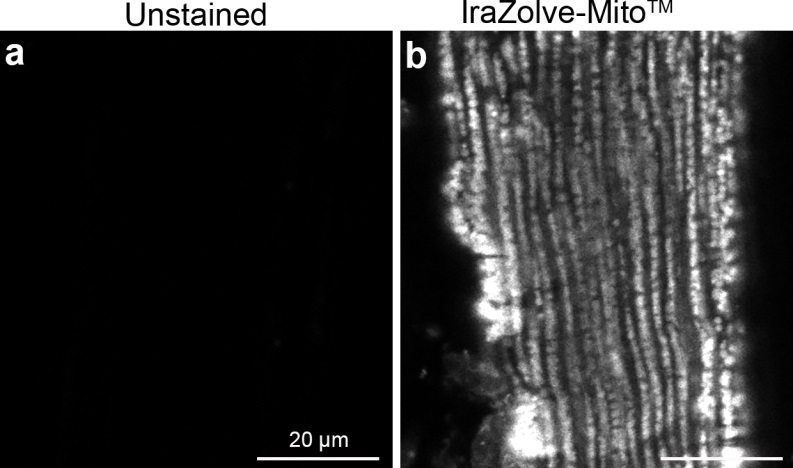


**Supplementary Figure 5|** Imaging IraZolve-Mito in skeletal muscle samples left panel control unstained tissue, right panel IraZolve-Mito stained tissue.
